# Supplementary material for: Levels of Plasma Endothelin-1, Circulating Endothelial Cells, Endothelial Progenitor Cells, and Cytokines after Cardiopulmonary Bypass in Children with Congenital Heart Disease: Role of Endothelin-1 Regulation
Source: Int J Mol Sci. 2024 Aug 15;25(16):8895. doi: 10.3390/ijms25168895 (PMC11354401; doi:10.3390/ijms25168895)
Supplement: Supplementary file 1 [file ijms-25-08895-s001.zip › ijms-3117815-supplementary.pdf]

Supplemental Materials

Table S1. Complementary Analysis.

Comparison of delta values according to the three study groups										
	control			CHD HPF			CHD LPF			
	median	p25	p75	median	p25	p75	median	p25	p75	p value
Delta CEC	-0.74	-1.61	0.55	-0.12	-1.21	0.49	-0.66	-4.43	0.23	0.3
Delta PEC	-212.74	-312.61	-151.49	-134.85	-303.56	-80.16	-235	-517.8	-74	0.5
Delta Endothelin	0.26	-2.27	0.54	-0.39	-0.84	-0.04	-0.4	-1.26	-0.06	0.1
Delta IL-8	-0.18	-7.47	0.6	-0.15	-2.87	0.37	-1.04	-5.18	0.22	0.4
Delta ilB	0	0	0	0	0	0	0	0	0	0.2
Delta Il6	-0.6	-8.82	0.36	-0.21	-6.9	0	-1.42	-20.03	0.11	0.6
Delta IL10	0	-2.61	0	0	-0.74	0.22	0	-1.35	0	0.4
Delta TNF alpha	0	0	0	0	0	0	0	0	0	0.3
Delta IL-12	0.23	-0.54	0.66	0	0	0.24	0	-0.56	0.04	0.2
Kruskall Wallis test										

CHD HPF= Congenital Heart Disease High Pulmonary Flow; CHD LPF=Congenital Heart Disease Low Pulmonary Flow

Linear correlation to explain the behavior of % of endothelial cells					
	Coefficients		Standarized Coefficients		p value
	B	Desv. Error	Beta	Desv. Error	
Endothelin 1 (second medition)	-2.42E-05	0	-0.54	-2.087	**0.046**
IL8 (second medition)	-4.46E-06	0	-0.067	-0.176	0.861
IL6 (second medition)	2.06E-05	0	0.229	0.874	0.39
IL10 (second medition)	0	0	-0.318	-0.92	0.366
IL12p70 (second medition)	-8.56E-05	0.001	-0.016	-0.078	0.939
% PEC 133 (second medition)	0.707	0.731	0.208	0.966	0.342
Weight (kg)	0	0	0.133	0.686	0.498
Surgery time (min)	3.96E-05	0	0.269	0.335	0.74
CPD (yes)	0.001	0.003	0.096	0.237	0.814
Aortic clamping	-1.65E-05	0	-0.097	-0.175	0.862
Constant	0.007	0.001		5.193	0

Linear correlation to explain the value of % EPC					
	Coefficients		Standarized Coefficients		p value
	B	Desv. Error	Beta	Desv. Error	
Endothelin 1 (second medition)	7.63E-06	0	0.58	2.896	0.007
IL8 (second medition)	8.50E-07	0	0.044	0.128	0.899
IL6 (second medition)	-4.04E-06	0	-0.153	-0.635	0.531
IL10 (second medition)	-2.85E-05	0	-0.105	-0.334	0.741
IL12p70 (second medition)	0	0	0.16	0.855	0.4
% PEC 133 (second medition)	3.74E-05	0	0.096	0.563	0.578
Weight (kg)	-1.41E-05	0	-0.326	-0.459	0.65
CPD (yes)	0.001	0.001	0.329	0.887	0.383
Aortic clamping	-1.28E-05	0	-0.257	-0.522	0.606
Type of CHD	-0.001	0.001	-0.169	-0.772	0.447
Constant	0.002			5.696	

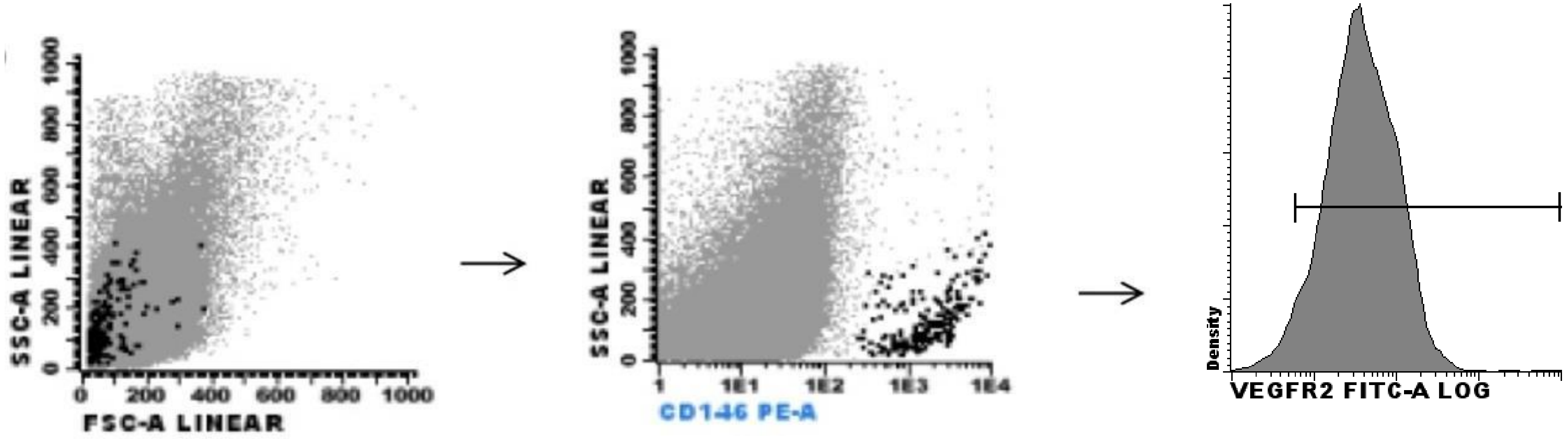

Figure S1. Analysis algorithm for identification of the endothelial cells before characterization with CD133 expression.
